# Hepatic FGF21 Deletion Improves Glucose Metabolism, Alters Lipogenic and *Chrna4* Gene Expression, and Enhances Telomere Maintenance in Aged Female Mice

**DOI:** 10.3390/ijms27010194

**Published:** 2025-12-24

**Authors:** Daniel Torres-Oteros, Mariano Nicola-Llorente, Héctor Sanz-Lamora, Albert Pérez-Martí, Pedro F. Marrero, Silvia Canudas, Diego Haro, Joana Relat

**Affiliations:** 1 Department of Nutrition, Food Sciences and Gastronomy, School of Pharmacy and Food Sciences, Food Torribera Campus, University of Barcelona, 08921 Santa Coloma de Gramenet, Spain; d.torres.oteros@ub.edu (D.T.-O.); nicola@ub.edu (M.N.-L.); h.sanz.lamora@ub.edu (H.S.-L.); albertperezmarti@gmail.com (A.P.-M.); pedromarrero@ub.edu (P.F.M.); silvia.canudas@ub.edu (S.C.); 2 Institute of Nutrition and Food Safety, University of Barcelona (INSA-UB), 08921 Santa Coloma de Gramenet, Spain; 3 Centro de Investigación Biomédica en Red de Fisiopatología de la Obesidad y Nutrición (CIBEROBN), Instituto de Salud Carlos III, 28029 Madrid, Spain; 4 Institute of Biomedicine, University of Barcelona (IBUB), 08028 Barcelona, Spain

**Keywords:** FGF21, females, ageing, metabolic health, telomere length, CHRNA4, liver

## Abstract

Fibroblast growth factor 21 (FGF21) is a key hormone for metabolic homeostasis under conditions such as obesity, aging and diabetes. While extensively studied in males, its role in female physiology remains poorly defined. This study evaluated the effects of hepatic FGF21 deletion in 12-month-old female mice using a liver-specific FGF21 knockout (FKO) model. FKO females exhibited reduced body weight and improved glucose tolerance, with no changes in circulating FGF21 levels. In the liver, RT-qPCR analysis showed that the expression of genes involved in de novo lipogenesis, including *Srebp1c*, *Fasn*, and *Scd1*, was downregulated, whereas markers of fatty acid uptake (*Cd36*) and β-oxidation (*Cpt1a*) were upregulated without alterations in hepatic triglyceride content and lower levels of serum adiponectin. Remarkably, telomere length in both liver and adipose tissue was preserved, indicating improved cellular aging. Hepatic transcriptomic analysis revealed a global downregulation of genes linked to cytoskeletal organization, immune processes and fibrosis. Among these, *Chrna4*, a hepatocyte-specific nicotinic acetylcholine receptor subunit implicated in protection against metabolic-associated steatohepatitis (MASH), was significantly reduced. These findings suggest that hepatic FGF21 deficiency in aged female mice promotes metabolic health by limiting pro-inflammatory and fibrotic pathways and preserving telomere integrity, with *Chrna4* emerging as a potential mediator.

## 1. Introduction

Fibroblast growth factor 21 (FGF21) is a hormone within the fibroblast growth factor (FGF) family, which comprises seven subfamilies with paracrine, autocrine, or endocrine functions. The FGF19 subfamily, including FGF19, FGF21, and FGF23, is distinguished by its endocrine activity [1,2]. FGF21 signals through a receptor complex formed by an FGF receptor (primarily FGFR1c) and the co-receptor β-klotho [3].

Under physiological conditions, FGF21 is primarily secreted by the liver but is also expressed in several tissues, including adipose tissue, muscle, and pancreas [4]. Its main role is to maintain metabolic homeostasis, becoming particularly relevant during metabolic stress conditions such as fasting, high-carbohydrate diets, ketogenic diets, and protein restriction [4,5,6]. However, FGF21 signaling is frequently impaired in metabolic disorders, including obesity, diabetes, and metabolic dysfunction-associated steatotic liver disease (MASLD) [6]. Obesity, in fact, is characterized by markedly elevated circulating FGF21 levels in obese mice, in rhesus monkeys fed a high-fat diet, and in overweight or obese humans. Yet, despite this induction, endogenous FGF21 appears largely ineffective in restoring metabolic balance. Conversely, pharmacological interventions successfully promote weight loss, improve glucose tolerance, and reduce circulating free fatty acids. This discrepancy led to the concept of an “FGF21-resistant state [7]” in obesity. However, this notion remains controversial, as the boundary between physiological and pharmacological effects of FGF21 and the precise mechanisms underlying its action are still not fully defined [8,9].

Beyond its metabolic role, FGF21 is also involved in the aging process [10]. Aging is an intrinsic, cumulative, and progressive phenomenon characterized by biochemical alterations, declining physiological capacity, disrupted energy metabolism, and changes in fat storage, often accompanied by impaired endocrine function [11]. Obesity and aging share common pathophysiological mechanisms at both molecular and systemic levels, including genomic instability, mitochondrial dysfunction, immune dysregulation, chronic inflammation, and telomere shortening. Telomeres, the protective DNA-protein structures at the ends of chromosomes, shorten progressively with each cell division and are particularly vulnerable to oxidative stress due to their guanine-rich sequence [12,13]. This attrition contributes to cellular senescence and is considered a hallmark of aging [14]. Telomere length (TL) is closely linked to metabolic processes such as inflammation, mitochondrial activity, and epigenetic regulation, making it a valuable biomarker of metabolic health and biological ageing [15]. These interconnected factors compromise tissue integrity and function, underscoring the close relationship between obesity and aging [16,17]. Furthermore, substantial evidence links metabolic disorders such as obesity, MASLD, and type 2 diabetes to accelerated aging [18].

Several studies have reported that circulating levels of FGF21 rise with age and that FGF21 resistance develops in both humans [19,20] and male rodents [21]. However, these findings remain inconsistent across sexes. Recent research reported lower FGF21 levels in older female mice compared to their younger counterparts [22].

FGF21 has been proposed as a modulator of healthspan and lifespan in mice [23,24], acting through mechanisms such as enhanced mitochondrial respiration and biogenesis, induction of autophagy, reduced insulin growth factor 1 (IGF-1) and growth hormone (GH) signaling, and anti-inflammatory effects [10]; pathways through which FGF21 may mitigate key hallmarks of ageing [25]. Despite this, most research has focused on males, leaving sex-specific effects largely unexplored. Females exhibit different metabolic responses to caloric restriction [26] protein restriction [27,28] and fasting [26,29], and FGF21 administration in obese mice produces sex-specific effects in the liver [30] and adipose tissue [31]. Moreover, physiological states unique to females, such as menstrual cycles and pregnancy, are known to elevate circulating FGF21 both in mice [32] and humans [33,34], with the liver acting as the primary source during these periods, at least in mice [32].

Considering the sex-specific differences in FGF21 regulation and the limited research on FGF21 function in females, the present study investigates the long-term metabolic impact of hepatic FGF21 deletion in aging female mice. By addressing this gap in knowledge, we aimed to define the role of FGF21 in female metabolic regulation and aging by assessing metabolic biomarkers, hepatic gene expression, mitochondrial and oxidative stress parameters, and telomere length.

## 2. Results

### 2.1. Lifelong Hepatic FGF21 Deficiency Reduces Body Weight and Improves Glucose Tolerance in Females

**Figure 1:**
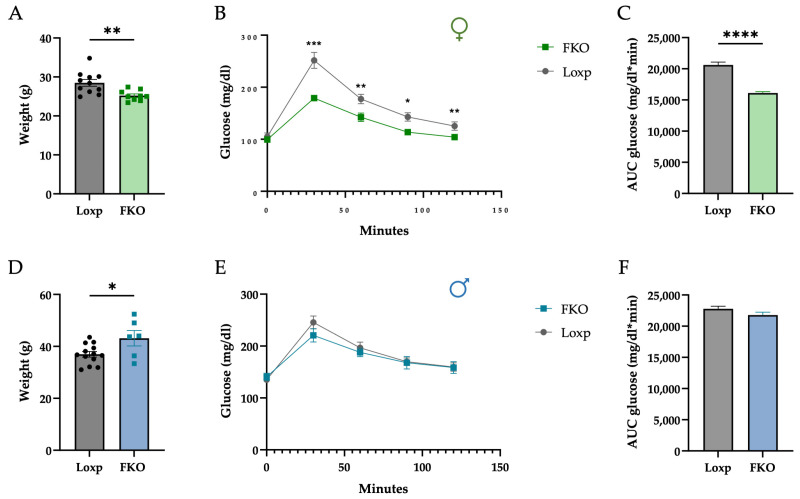
Lack of hepatic FGF21 reduces body weight and improves glucose tolerance exclusively in females. Body weight of female mice (**A**) and male mice (**D**) at the end of the study. Plasma glucose levels in females (**B**) and in males (**E**) following an intraperitoneal glucose injection (1.5 g/kg body weight) during a glucose tolerance test (GTT). Area under the curve (AUC) for plasma glucose in females (**C**) and in males (**F**). Data are presented as mean ± SEM. * *p* < 0.05; ** *p* < 0.01; *** *p* < 0.001; **** *p* < 0.0001 (Student’s *t*-test). Female LoxP: n = 11; FKO: n = 9. Male LoxP: n = 13; FKO: n = 6.

Female mice lacking hepatic FGF21 (FKO) displayed significantly lower body weight (Figure 1A), and improved glucose tolerance compared to LoxP controls (Figure 1B,C). Regarding liver weight, although absolute liver mass was significantly lower in FKO animals, this difference disappeared when values were normalized to total body weight. These effects were absent in males (Figure 1D–F), indicating a clear sex-specific response.

Based on these results, all subsequent analyses were directed toward elucidating the metabolic and molecular consequences of hepatic FGF21 deletion in female mice.

**Figure 2:**
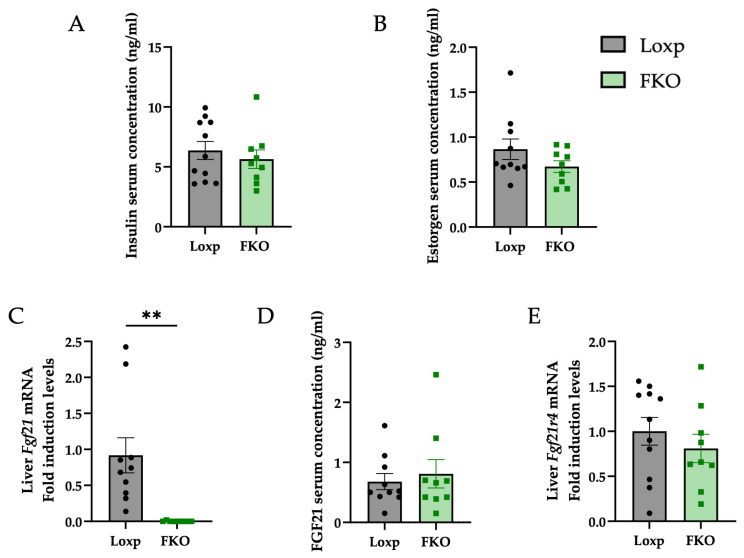
Hepatic FGF21 deletion does not alter circulating FGF21, insulin, or estrogen levels. (**A**) Basal insulin serum concentration (pg/mL). (**B**) Basal estrogen serum concentration (pg/mL). (**C**) Hepatic *Fgf21* mRNA expression. (**D**) FGF21 serum levels (pg/mL). (**E**) Hepatic expression of *Fgf21 receptor 4* (*Fgf21r4*). All data are presented as mean ± SEM. ** *p* < 0.01 and *p*-values were determined by using a Student’s *t*-test. LoxP: n = 11; FKO: n = 9. For ELISA: LoxP: n = 10; FKO: n = 9.

Serum insulin concentrations were measured to determine whether the improved glucose response was associated with changes in insulin levels; however, no significant differences were detected between groups (Figure 2A). Considering that FGF21 is a hormone, the possibility that its absence could influence other hormones, such as estrogens, which contribute to sexual dimorphism, was evaluated. Nonetheless, serum estrogen levels remained unchanged in FKO females compared to control group (Figure 2B).

Although the liver is the primary source of circulating FGF21 and liver-specific *Fgf21* knockout (FKO) mice exhibited complete absence of hepatic *Fgf21* expression (Figure 2C), basal plasma levels did not differ between groups (Figure 2D), suggesting a compensatory secretion form extrahepatic tissues. Furthermore, hepatic expression of *Fgf21R4*, a membrane receptor for FGF21, showed no significant variation (Figure 2E).

### 2.2. FKO Females Exhibit Longer Telomeres in Hepatocytes and Adipocytes

**Figure 3:**
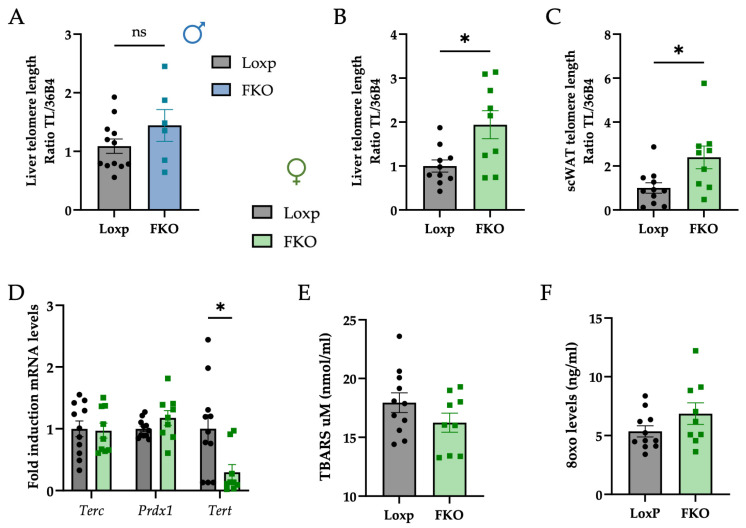
Hepatic FGF21 deletion preserves telomere length in females without affecting oxidative stress markers and reduces telomerase mRNA expression. (**A**) Telomere length (ratio of telomere to 36b4 reference gene) in (**A**) male liver, (**B**) female liver, (**C**) female scWAT. (**D**) Relative mRNA levels of genes related to telomere regulation: *Terc* (*Telomerase RNA component*), *Prdx1* (*Peroxiredoxin 1*) and *Tert* (*Telomerase reverse transcriptase*). (**E**) Malondialdehyde (MDA) levels in livers of mice measured fluorometrically by the TBARS assay. (**F**) 8-hydroxy-2-deoxyguanosine (8oxo) levels in livers of mice measured with colorimetric assay. All data are presented as mean ± SEM. ns: non-significant; * *p* < 0.05 and *p*-values were determined by using a Student’s *t*-test. Female LoxP: n = 11; FKO: n = 9. Male LoxP: n = 13; FKO: n = 6.

In light off the observed differences in body weight and glucose tolerance, telomere length was assessed as an aging-related parameter. Analysis revealed no differences in telomere length among male mice (Figure 3A). However, FKO females displayed significantly longer telomeres in both liver and subcutaneous white adipose tissue (scWAT) compared to LoxP controls (Figure 3B,C). These findings suggest a sex-specific effect of the FKO genotype, potentially conferring enhanced cellular longevity or resilience in female metabolic tissues.

To explore potential mechanisms, underlying the increased telomere length observed in FKO females, the hepatic expression of key genes involved in telomere regulation was examined. No significant changes were detected in the mRNA levels of *telomerase RNA component* (*Terc*) or *peroxiredoxin 1* (*Prdx1*), while *telomerase reverse transcriptase* (*Tert*) was significantly downregulated in FKO females (Figure 3D).

Because *Tert* is widely considered the rate-limiting component of telomerase activity in somatic cells and telomere length was increased in FKO females, it was hypothesized that reduced oxidative stress could contribute to telomere preservation. Lipid peroxidation was measured using the Thiobarbituric Acid Reactive Substances (TBARS) test, while DNA oxidation was evaluated through 8-hydroxy-2-deoxyguanosine (8oxo) levels. No significant differences were detected between groups for both parameters (Figure 3E,F).

### 2.3. Hepatic FGF21deficiency Suppresses Lipogenic Genes and Enhances Fatty Acid Uptake and b-Oxidation Markers in the Liver

Consistent improvements in glucose tolerance and telomere length maintenance observed in FKO female mice prompted further investigation into the metabolic consequences of hepatic FGF21 deficiency. Given the crucial role of FGF21 in regulating lipid and glucose homeostasis, its absence in the liver was evaluated with respect to different key metabolic pathways [35].

The analysis of lipid metabolism revealed a significant downregulation of genes related to de novo lipogenesis, including *sterol regulatory element-binding transcription factor 1* (*Srebp1c*), *fatty acid synthase* (*Fasn*), and *stearoyl-Coenzyme A desaturase 1* (*Scd1*). Additionally, downward trend in the expression of *Carbohydrate responsive element binding protein* (*Chrebp*) was also noted in the FKO group relative to LoxP controls, although statistical significance was not achieved.

**Figure 4:**
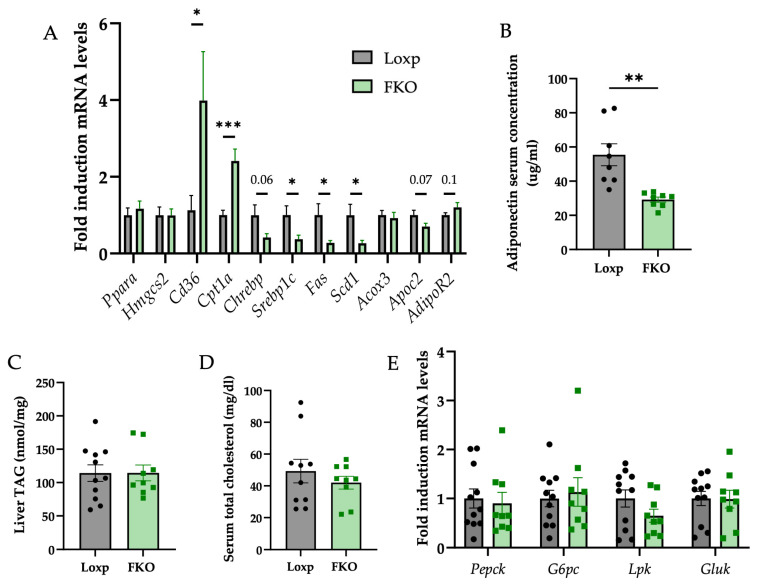
Gene expression changes related to lipid and glucose metabolism in the liver of FKO mice, along with serum adiponectin levels. (**A**) Relative mRNA levels of genes related to lipid metabolism: *Pparα* (*Peroxisome proliferator activated receptor alpha*), *Hmgcs2* (*3-hydroxy-3-methylglutaryl-Coenzyme A synthase 2*), *Cd36* (*CD36 molecule*), *Cpt1a* (*Carnitine palmitoyltransferase 1a*), *Chrebp* (*MLX interacting protein-like*), *Srebp1c* (*Sterol regulatory element binding transcription factor 1*), *Fasn* (*Fatty acid synthase*), *Scd1* (*Stearoyl-Coenzyme A desaturase 1*), *Acox3* (*Acyl-Coenzyme A oxidase 3*), *Apoc2* (*Apolipoprotein C2 like*) *and AdipoR2* (*Adiponectin Receptor 2*). (**B**) Adiponectin serum levels (μg/mL). (**C**) Liver Triacylglycerol (TAG) levels (µmol/mg). (**D**) Total cholesterol serum levels (mg/dL). (**E**) Relative mRNA levels of genes related to gluconeogenesis: *Pepck* (*Phosphoenolpyruvate carboxykinase 1*) and *G6pc* (*Glucose-6-phosphatase*); and *glycolysis: Lpk* (*Pyruvate kinase*) and *Gluk* (*Glucokinase*). All data are presented as mean ± SEM. * *p* < 0.05; ** *p* < 0.01; *** *p* < 0.001 and *p*-values were determined by using a Student’s *t*-test. LoxP: n = 10; FKO: n = 9. For adiponectin ELISA: LoxP: n = 8; FKO: n = 8.

In contrast, genes associated with fatty acid uptake and mitochondrial β-oxidation were upregulated. Specifically, *cluster of differentiation 36* (*Cd36*), which facilitates fatty acid import into cells and *carnitine palmitoyltransferase 1a* (*Cpt1a*), the enzyme that catalyzes the formation of acyl-carnitines enabling the transport of long-chain fatty acids into the mitochondrial matrix, exhibited increased expression in FKO livers (Figure 4A). No significant changes were detected in the mRNA levels of genes involved in ketogenesis.

As these transcriptional changes are downstream of PPARα signalling, serum adiponectin levels, an upstream regulator of PPARα [36] were quantified and found to be reduced in FKO group (Figure 4B). However, hepatocyte sensitivity to adiponectin, assessed via receptor expression, showed a modest upward trend (Figure 4A). Regarding cholesterol metabolism, *apolipoprotein C2-like (Apoc2)* expression exhibited a decreasing trend in the FKO group compared to the LoxP group (Figure 4A). To determine whether these transcriptional changes affected the lipid profile, hepatic triglyceride (TG) and serum cholesterol levels were measured. No significant differences were observed in either TG or cholesterol levels between groups (Figure 4C,D).

Finally, considering the improved glucose tolerance observed in FKO mice, hepatic glucose metabolism was evaluated. Expression levels of gluconeogenic genes, *phosphoenolpyruvate carboxykinase 1 (Pepck)* and *glucose-6-phosphatase (G6pc)* and glycolytic genes, *pyruvate kinase (Lpk)* and *glucokinase (Gluk)*, remained unchanged (Figure 4E), indicating that hepatic glucose flux may not be directly influenced by FGF21 deficiency.

**Figure 5:**
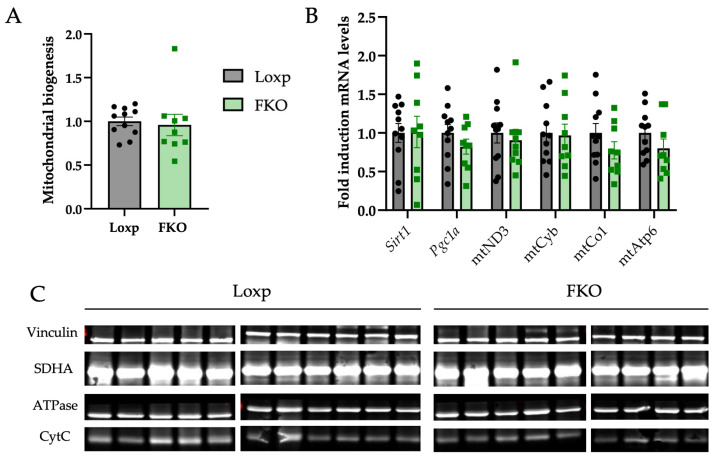
Assessment of mitochondrial biogenesis and expression of genes and proteins related to oxidative phosphorylation (OXPHOS) in the liver of FGF21 knockout (FKO) mice. (**A**) Ratio of the mitochondrial DNA content, calculated as the genomic abundance of *Cox1* (*Cytochrome c oxidase subunit I*) relative to *Hbbt2* (*Beta-globin*). (**B**) Relative mRNA expression of *Sirt1* (*Sirtuin 1*), *Pgc1a* (*Peroxisome proliferative activated receptor gamma coactivator 1 alpha*), *mtND3* (*NADH dehydrogenase 3*), *mtCytb* (*cytochrome b*), *mtCo1* (*cytochrome c oxidase I*) and *mtAtp6* (*ATP synthase 6*). (**C**) Western blot analysis of mitochondrial proteins from OXPHOS complexes: SDHA (Complex II), ATPase (Complex V) and CytC (Complex IV). All data are presented as mean ± SEM. *p*-values were determined by using a Student’s *t*-test. Loxp: n = 10; FKO: n = 9.

FGF21 has also been implicated in the regulation of mitochondrial biogenesis, primarily through activation of peroxisome proliferator-activated receptor-γ coactivator 1α (PGC1a) via AMP-activated protein kinase (AMPK) and sirtuin 1 (SIRT1) signaling pathways [37]. However, no differences in mitochondrial biogenesis were detected between groups (Figure 5A). Similarly, no significant changes were observed in the expression levels of *Sirt1* and *Pgc1a* (Figure 5B). Despite transcriptional changes observed in genes regulating lipid oxidation, no significant changes were found in components of the mitochondrial electron transport chain at either the mRNA (Figure 5B) or protein level (Figure 5C).

### 2.4. Hepatic FGF21 Deficiency Downregulates Genes Related to Cytoskeletal Organization and Immune System in the Liver, Potentially Mediated by ChRNA4

**Figure 6:**
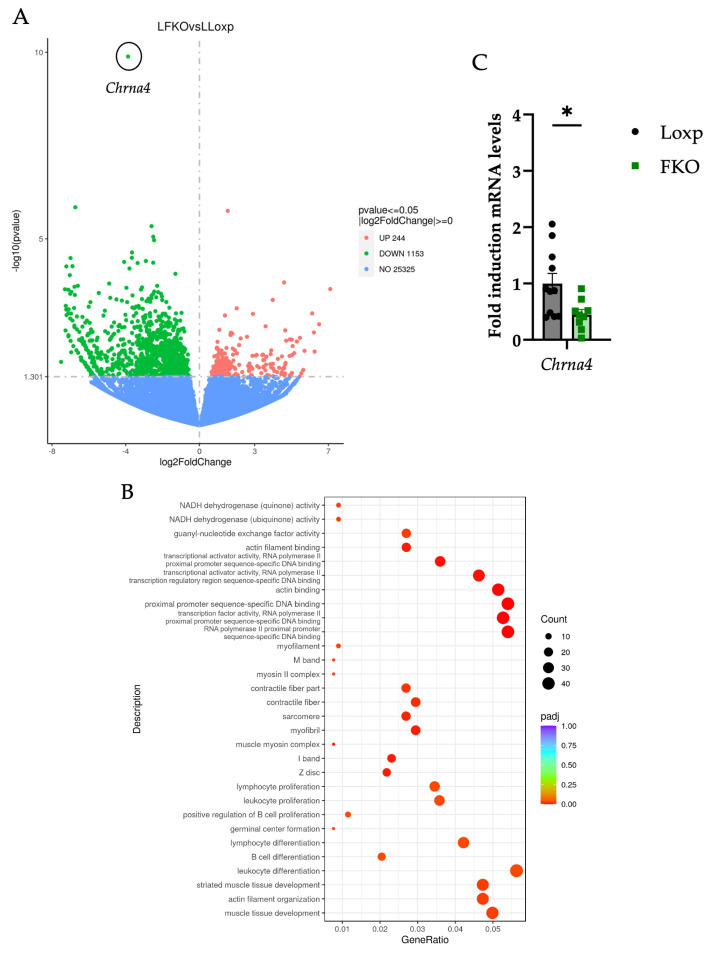
Transcriptomic analysis of liver tissue reveals downregulation of genes associated with fibrosis, cytoskeletal organization and immune function in FKO mice. (**A**) Volcano plot illustrating differential gene expression (FC > 1.3 and *p* < 0.05) between LoxP and FKO groups. Red circles indicate upregulated genes; green circles indicate downregulated genes. *Chrna4* (*Cholinergic receptor nicotinic alpha 4*) gene was the one that showed the most significant differential expression between groups. Liver RNA-seq was performed using pooled liver samples from LoxP and FKO mice. (**B**) Gene Ontology (GO) enrichment of downregulated genes. (**C**) Validation of *Chrna4* downregulation by qPCR in liver tissue. All data are presented as mean ± SEM. * *p* < 0.05, *p*-values were determined by using a Student’s *t*-test. LoxP: n = 10; FKO: n = 9.

To further characterize the effects of hepatic FGF21 deficiency in aged female mice, transcriptomic profile of liver tissue was performed using RNA sequencing (RNA-seq). The complete dataset is available on request. Transcriptomic analysis revealed a global downregulation of liver gene expression in the FKO group, with the *cholinergic receptor nicotinic alpha 4* (*Chrna4*) identified as the most significantly downregulated gene (Figure 6A).

Gene Ontology (GO) enrichment analysis of the downregulated genes indicated a significant overrepresentation of terms related to cytoskeletal organization, including actin filament organization, sarcomere, contractile fibers, and Z disc components (Figure 6B). Additionally, GO terms associated with the immune system processes, such as lymphocyte proliferation, B cell differentiation, and leukocyte differentiation were enriched, suggesting a reduction in immune activity. Several terms also pointed to fibrogenesis-related processes, including muscle tissue development and components linked to myofibroblast activation (e.g., myofibril, muscle myosin complex, M band), which are commonly associated with the hepatic stellate cell activation [38,39].

Consistent with these GO-enriched categories, the hepatic RNA-seq dataset revealed a coordinated reduction in transcripts linked to inflammatory and fibrogenic activity. Several genes associated with canonical profibrotic pathways exhibited lower expression in FKO livers, including *Smad1*, a downstream mediator of TGF-β signaling involved in fibrogenic responses [40], *Mef2c* and *Mef2b*, transcription factors implicated in hepatic stellate cell activation [41], and *Fbn1*, an extracellular matrix component associated with fibrotic remodeling [42]. In parallel, the chemokine *Cxcl10*, which promotes macrophage M1 polarization and contributes to inflammatory progression in steatotic liver disease, was also downregulated [43].

Downregulation of *Chrna4* mRNA expression was validated by qPCR (Figure 6C). CHRNA4 is a subunit of the nicotinic acetylcholine receptors (nAChRs) specifically expressed in hepatocytes. A recent study has demonstrated that *Chrna4* expression is elevated in metabolic dysfunction-associated steatohepatitis (MASH) and that its activation promotes pro-inflammatory cytokine production contributing to the progression of liver inflammation. Conversely, inhibition or downregulation of CHRNA4 has been shown to confer protection against MASH pathogenesis [44]. These findings suggest that hepatic FGF21 deficiency may modulate immune regulation, cytoskeletal integrity, and fibrogenic pathways, potentially through mechanisms involving CHRNA4.

## 3. Discussion

FGF21 is widely recognized as a key metabolic regulator that modulates ageing-related processes, with its secretion typically linked to improved capacity to maintain energy homeostasis [10,45]. However, the specific role of hepatic FGF21 in aged females has been less well defined.

In the present study, aged liver-specific FGF21 knockout (FKO) females displayed a reduction in body weight and improved glucose tolerance compared with LoxP controls, while aged FKO males exhibited higher body weight and no change in glucose tolerance (Figure 1). These findings support a sex-specific influence of hepatic FGF21 during ageing.

Despite complete deletion of hepatic *Fgf21* expression (Figure 2C), basal circulating FGF21 levels remained unaltered (Figure 2D). This result aligns with the view that extrahepatic tissues can compensate for the absence of liver-derived FGF21 under non-stressed conditions. Other tissues, including adipose tissue, muscle, and pancreas, can secrete FGF21 in the absence of stress. Typically, hepatic FGF21 production is induced under metabolic challenges such as fasting, high-fat or low-protein diets, and metabolic syndromes, including MAFLD [4,6,46,47,48].

In females, FGF21 production is also linked to reproductive physiology, with reported fluctuations during the menstrual/estrous cycle and pregnancy [32,49].

Although circulating FGF21 was not monitored throughout the estrous cycle in FKO females, the absence of liver-derived FGF21 is expected to abolish the physiological cyclical increases in this hormone, which may partly explain the sex-specific phenotype observed in knockout females compared with males. In addition, FGF21 has been linked to estrogen regulation and reproductive function [49,50,51]. In the present study, estrogen levels did not differ between FKO and LoxP females (Figure 2B); however, previous studies have reported that females with a liver-specific deletion of FGF21 maintain estrous cyclicity for longer periods than their wild-type counterparts [52], suggesting a role for FGF21 in reproductive ageing. Moreover, although circulating FGF21 concentrations and hepatic receptor expression were similar between LoxP and FKO females, these static measurements do not reflect the temporal dynamics of FGF21 signaling across the estrous cycle. We therefore hypothesize that the hepatic effects observed result from the lifelong absence of FGF21 signaling in extrahepatic tissues of knockout females, particularly during periods when circulating FGF21 levels would normally be elevated in wild-type animals.

Importantly, age-related changes in FGF21 expression are context dependent: hepatic expression decreases in male mice [53], while circulating plasma levels of FGF21 increase in humans [20,54].

Telomere length is influenced by multiple factors, including oxidative and metabolic stress, as well as cellular turnover, and its progressive shortening is widely recognized as a hallmark of cellular ageing [55,56]. In the present study, telomere preservation emerged as one of the most striking sex-specific features: aged FKO females exhibited significantly longer telomeres in hepatic and adipose tissues compared with controls, despite a marked downregulation *Tert*, which is the catalytic subunit of telomerase (Figure 3A–D). *Tert* is a reverse transcriptase whose primary role is to elongate telomeres. In hepatocytes, telomerase activity remains limited, reflecting their restricted replicative capacity. It is important to emphasize, however, that telomere length is more indicative of cumulative replicative and oxidative stress than of *Tert* expression itself [57]. Thus, the elongated telomeres observed in aged FKO females are likely the result of reduced lifelong telomere attrition and alternative maintenance mechanisms, rather than direct telomerase-mediated elongation.

One plausible explanation is a reduction in cellular turnover or replicative stress in FKO females, which could result in decreased telomere attrition over time. In this context, telomeres are preserved not because they are actively elongated, but because they are less frequently eroded. Although no significant differences were observed in markers of oxidative damage, namely lipid peroxidation (TBARS) and DNA oxidation (8oxo) (Figure 3E,F), other unmeasured antioxidant pathways, stress-response systems, or genomic stability mechanisms may contribute to telomere protection. These findings point to a potentially more quiescent or metabolically balanced cellular environment in FKO females, favoring telomere integrity independently of telomerase upregulation.

Sex differences in telomere length have been previously reported, with women generally exhibiting longer telomeres than men [58,59,60]. Estrogen has been proposed to play a protective role by modulating oxidative stress and enhancing antioxidant defenses, effects that may persist beyond reproductive age. Moreover, lifestyle and metabolic factors may interact with sex-specific regulatory mechanisms to influence telomere dynamics. Although oxidative stress markers did not differ between groups in our study, the longer telomeres observed in aged FKO females suggest that other mechanisms unrelated to measured oxidative stress parameters may contribute to this phenotype.

These findings point to a complex interplay between reduced cellular turnover, diminished replicative and oxidative stress, and the possible activation of alternative antioxidant or genomic maintenance mechanisms. Such adaptations may foster a more stable metabolic and redox environment, thereby promoting telomere preservation independently of canonical telomerase activity. Taken together, these results suggest that hepatic FGF21 deficiency modulates aging not only through metabolic adaptations but also by influencing telomere biology in a sex-dependent manner. The enhanced telomere integrity observed in FKO females, despite reduced *Tert* expression, underscores the complexity of telomere regulation and highlights the potential for non-canonical mechanisms of telomere maintenance under conditions of altered metabolic signaling.

Metabolism is a key determinant of telomere regulation and cellular stress response, and FGF21 exerts systemic effects, particularly in adipose tissue, where it regulates lipid and glucose homeostasis and promotes adipokine secretion [61]. In the present study, FKO females showed marked suppression of lipogenic genes, alongside upregulation of fatty acid uptake and oxidation markers in the liver (Figure 4A). These adaptations occurred without significant changes in hepatic triglyceride or cholesterol content (Figure 4C,D), and without alterations in glycolytic or gluconeogenic genes (Figure 4E), pointing to a selective reprogramming of hepatic lipid metabolism.

Interestingly, these transcriptional changes were associated with reduced serum adiponectin (Figure 4B). Adiponectin is a key mediator of inter-organ communication [62], and its decline would typically be expected to reduce hepatic responsiveness. However, the liver of FKO females showed enhanced PPARα signaling, with a trend toward increased adiponectin receptor expression (Figure 4A). Adiponectin is known to activate AMPK and increase PPARα protein abundance via AdipoR1/2 and APPL1 [62]. Thus, despite reduced circulating adiponectin, the liver appeared to mount a stronger PPARα response. One possible explanation is the greater availability of endogenous PPARα ligands from adipose depots other than scWAT [63], where no changes in mRNA expression were detected (Figure S1). Activation of lipolysis in adipocytes releases fatty acids into the circulation, which can subsequently regulate hepatic gene expression [64]. Another possibility is compensatory activation of PPARα in response to the absence of FGF21, a downstream target of this pathway [65], as a mechanism to restore FGF21 expression and maintain homeostasis.

In this context, it is worth mentioning that previous reports on the relationship between aging and adiponectin levels are inconsistent, with some studies describing a decline, others no changes and others an increase [65,66,67,68]. These discrepancies highlight the complex and context-dependent regulation of adiponectin during aging.

Enhanced PPARα activity would be expected to promote fatty acid uptake and β-oxidation, consistent with the upregulation of *Cd36* and *Cpt1a*. Nevertheless, mitochondrial content and expression of oxidative phosphorylation genes and proteins were unchanged (Figure 5A–C), suggesting that β-oxidation was increased without broad alterations in mitochondrial biogenesis or electron transport chain function. Together with reduced de novo lipogenesis and stable hepatic triglyceride levels, these results suggest that the liver of FKO females relies more heavily on circulating fatty acids to meet its energetic demands.

Beyond its metabolic role, FGF21 also functions as a stress-response hormone with established anti-inflammatory properties. Under pharmacological treatment or stress-induced production, FGF21 typically reduces NF-κB activation, promotes macrophage polarization toward the anti-inflammatory M2 phenotype, and limits immune cell infiltration in the liver [69,70,71,72,73,74]. In contrast, under basal ageing conditions, gene expression analysis revealed a broad downregulation of genes related to immune cell differentiation and proliferation in FKO mice (Figure 6B). These findings suggest that the lifelong absence of hepatic FGF21 in non-stressed, otherwise healthy females elicits effects distinct from the acute or pharmacological actions of FGF21.

Moreover, genes from pathways related to cytoskeletal remodeling were also downregulated (Figure 6B), a hallmark of hepatic stellate cell (HSC) activation and fibrosis [75,76]. These changes in the expression may therefore indicate reduced fibrogenic potential in the FKO group.

Among the downregulated genes, *Chrna4* emerged as the most significant in FKO females (Figure 6A–C). *Chrna4* encodes a nicotinic acetylcholine receptor subunit expressed in hepatocytes, which mediates immune–hepatocyte communication by responding to acetylcholine released from immune cells [77]. Its activation promotes pro-inflammatory cytokine release and contributes to MASH progression, while its inhibition has been shown to confer protection against inflammation and fibrosis [44]. Thus, decreased *Chrna4* levels in FKO livers may attenuate acetylcholine-driven cytokine production and limit immune cell–hepatocyte crosstalk, thus suggesting a protective effect against age-related inflammatory and fibrotic liver changes.

Collectively, these findings indicate that, in aged females, the absence of hepatic FGF21 may paradoxically protect against inflammatory and fibrotic remodeling, highlighting a context- and sex-specific divergence from the canonical anti-inflammatory role of FGF21 under stress.

Taken together, these results highlight the complexity of FGF21 biology in ageing females. While FGF21 is generally regarded as beneficial for metabolic homeostasis, its lifelong absence in the liver under non-stress conditions promoted favorable metabolic and cellular adaptations, including reduced body weight, improved glucose tolerance, preserved telomeres, suppression of lipogenesis, enhanced PPARα activity, and reduced immune and fibrotic signaling. These outcomes appear to be context- and sex-dependent. Indeed, while FGF21 administration has demonstrated beneficial effects in males [78], its impact in females would not be the same. For instance, Makarova et al. demonstrated that the administration of FGF21 to obese mice resulted in reduced insulin levels and liver steatosis in males but that these effects were blunted in females [30], but also that FGF21 administration beneficially impact on mice of both sexes but inducing female-specific activation of gene expression in WAT [31].

Beyond these findings, additional evidence supports a broader sexual dimorphism in FGF21 biology. Chaffin et al. showed that FGF21 reduced hepatic triglyceride accumulation in obese male mice but not in females, indicating that the adiponectin–FGF21–liver axis operates differently between sexes [79]. In addition, it has been reported that the hepatoprotective role of FGF21 in females depends on intact estrogen signaling, as ovariectomy abolished FGF21-mediated resistance to diet-induced steatosis unless estrogen was restored [80].

Together, these studies indicate that females rely on distinct transcriptional, hormonal and inter-organ communication pathways to regulate FGF21-related metabolic responses, particularly under physiological (non-stress) conditions.

Moreover, it has been described that females respond differently to nutritional challenges where FGF21 activity is prominent, such as caloric and protein restriction diets or fasting [26,27,28,29,81].

In this context, the phenotype observed in aged FKO females in our study, improved glucose tolerance, selective reprogramming of hepatic lipid metabolism, reduced inflammatory signaling and enhanced telomere preservation, suggests that the absence of hepatic FGF21 unmasks female-specific compensatory mechanisms that remain silent or are fundamentally different in males. These results further reinforce the importance of including female models when investigating metabolic and ageing-related processes, as conclusions derived from male-only studies may fail to capture key regulatory mechanisms that are operative in females.

This sexual dimorphism is consistent with broader physiological differences between males and females. Women generally exhibit greater resistance to developing metabolic diseases when exposed to a high-fat diet compared to men [82], primarily due to their enhanced capacity to store fat more efficiently to meet reproductive demands. Conversely, men are more susceptible to developing MASLD and other lipid-related pathologies [83]. These differences are mediated, at least in part, by distinct growth hormone (GH) secretion patterns, which modulate STAT5B activity. This signaling cascade regulates transcription factors such as Bcl6, a repressor of female-biased genes, and Cux2, which promotes the expression of female-specific genes in hepatocytes [84].

Limitations: The current study was limited by the absence of longitudinal monitoring of FGF21 and sex hormones across the estrous cycle and pregnancy, which precludes establishing direct links between reproductive physiology and the observed female-specific phenotype. Only scWAT was analyzed, restricting conclusions about adipose–liver communication, and mechanistic insight into PPARα activation was inferred without direct measurement of endogenous ligands or functional perturbation. Finally, the absence of stressor challenges (e.g., dietary or inflammatory) constrains interpretation to basal conditions. Finally, the interpretation of sex-dependent effects should be made with caution, as male and female mice were not exactly age-matched. This difference represents a limitation of the study design and may influence the magnitude of the observed telomere maintenance.

## 4. Materials and Methods

### 4.1. Mice Procedures

#### 4.1.1. Mice Housing

All animal procedures were approved by the Animal Ethics Committee of the University of Barcelona. Liver-specific FGF21 knockout (FKO) mice were previously generated by our group [48]. Briefly, Fgf21^loxP^ mice (Fgf21^tm1.2Djm/J^) that have Fgf21 flanked by two *loxP* sites (Jackson Laboratory, Sacramento, CA, USA) were crossed with Albumin-cre (Tg(Alb1-cre)1Dlr/J) mice (kindly provided by Dr. A. Zorzano). The latter express the CRE recombinase enzyme under control of albumin promoter/enhancer elements, thus allowing liver-specific gene deletions. FGF21 LoxP littermates were used as controls. One-year-old C57BL/6J female mice (LoxP n = 11 and FKO n = 9) were analyzed first. Subsequently, based on initial observations in weight, glucose tolerance and telomere length; male mice of the age available in the colony (1.5 years) were included to determine whether similar changes occurred in males (LoxP n = 13 and FKO n = 6). As the study proceeded in two sequential phases, the male and female groups were not age-matched at the time of analysis. Mice were housed in a temperature-controlled room (22 ± 1 °C) under a 12/12 h light/dark cycle, with ad libitum access to standard chow diet and filtered tap water.

#### 4.1.2. Glucose Tolerance Test (GTT)

A glucose tolerance test (GTT) was performed one week prior to euthanasia. Mice were fasted for 6 h (from 8 a.m. to 2 p.m.) in a clean cage with ad libitum access to water before the glucose injection. Body weight was recorded before the procedure. A glucose solution (1.5 mg glucose/g body weight) was administered intraperitoneally, and blood glucose levels were measured at 0, 30, 60, 90, and 120 min post-injection using a glucometer (Glucocard SM, Menarini, Florence, Italy). Blood samples were collected from the tail vein.

#### 4.1.3. Tissue and Serum Collection

Mice were euthanatized by cervical dislocation. Blood was collected via intracardiac puncture, and serum was obtained by clotting and centrifugation (1500 rpm, 30 min). Liver and other tissues were isolated and immediately snap-frozen at −80 °C for subsequent analysis.

### 4.2. Thiobarbituric Acid Reactive Substances (TBARS) Assay

Lipid peroxidation was quantified by measuring malondialdehyde (MDA) levels in liver tissue (25 mg) using a TBARS assay (Abnova, Taipei City, Taiwan), following the manufacturer’s instructions. Assays were run in duplicate, with MDA standards used to generate a calibration curve.

### 4.3. ELISA Assays

#### 4.3.1. DNA Damage Competitive ELISA

Serum 8-hydroxy-2-deoxyguanosine (8-OHdG), a marker of DNA oxidation, was quantified using the DNA Damage Competitive ELISA kit (EIADNAD, Thermo Fisher Scientific, Waltham, MA, USA). Serum samples were diluted 1:10 and analyzed in duplicate. Absorbance at 450 nm was measured with a Fluoroskan Ascent ELISA plate reader (Thermo Fisher Scientific, Barcelona, Spain).

#### 4.3.2. Mouse Estrogen Competitive ELISA

Serum estrogen levels were measured using a Competitive ELISA kit (MOFI01442, AssayGenie, Dublin, Ireland). Serum samples were diluted 1:2 and analyzed in duplicate. Absorbance at 450 nm was recorded with a Fluoroskan Ascent ELISA plate reader (Thermo Fisher Scientific, Spain).

#### 4.3.3. Mouse Insulin and Fgf21 Sandwich ELISA

Serum insulin (MOFI00142, AssayGenie) and FGF21 (MOFI00141, AssayGenie) levels were measured using Sandwich ELISA kits. Serum samples were diluted 1:5 for insulin and 1:10 for FGF21 and analyzed in duplicate. Absorbance at 450 nm was measured with a Fluoroskan Ascent ELISA plate reader (Thermo Fisher Scientific, Spain). 

### 4.4. Serum Cholesterol and Hepatic Triglycerides

Total serum cholesterol was quantified using a Cholesterol Quantification Kit (MAK043-1KT, Sigma Aldrich, Saint Louis, MO, USA). Serum samples were diluted 1:20 and fluorescence was measured at an excitation wavelength of 535nm and an emission wavelength of 570nm with a Fluoroskan Ascent ELISA plate reader (Thermo Fisher Scientific, Spain).

Hepatic triglyceride content was measured in homogenized liver tissue (100 mg of liver tissue in 1 mL of 5% Nonidet P40 solution (A1694,0250, PanReac AppliChem, Montcada i Reixac, Barcelona, Spain). using the Triglyceride Quantification Colorimetric Kit (MAK266-1KT, Sigma Aldrich, USA). Absorbance at 570 nm was measured with a Fluoroskan Ascent ELISA plate reader (Thermo Fisher Scientific, Spain).

### 4.5. RNA Isolation and Retro-Transcription Quantitative PCR (RT-qPCR)

Total RNA was isolated from liver and scWAT using TRI Reagent™ (AM9738, Invitrogen, Waltham, MA, USA) and following the manufacturer’s instructions. Tissue homogenization in TRI Reagent™ correctly was performed with a pellet pestle (Z359971, Sigma-Aldrich, Saint Louis, MO, USA). After RNA extraction, all samples were treated with DNase I (K2981, Thermo Scientific), quantified and assessed for purity using a BioDrop spectrophotometer (Harvard Bioscience™, Holliston, MA, USA).

cDNA was synthesized from 1.5 μg (liver) or 1 μg (scWAT) RNA using a High-Capacity cDNA Reverse Transcription Kit (10400745, Applied Biosystems, Waltham, MA, USA) and following the manufacturer’s instructions. qPCR was performed on a CFX96 Touch Real-Time PCR Detection System with SYBR™ Select Master Mix (13226529, Applied Biosystems) using 1:50 diluted samples and a relative standard curve from a pool of all samples. All samples were run in duplicate. Reference genes were B2m and 18S (liver) or B2m and β-actin (scWAT), and a bestkeeper was calculated. The primer sequences used are shown in Table S1.

### 4.6. RNA-Seq Analysis

RNA-seq was performed on pooled liver samples (2–3 animals per pool). Library preparation and sequencing were conducted by Novogene (Illumina PE150, Cambridge, UK). Quality control, read filtering, and genome alignment (using the Mus musculus reference genome (GRCm38/mm10)) were performed using Novogene’s standard bioinformatic pipeline. Gene-level quantification was obtained from aligned reads, and differential expression analysis was performed in R using DESeq2 (FDR < 0.05). Gene Ontology (GO) and KEGG pathway enrichment analyses were conducted in R version 4.4.1 using validated enrichment tools with Benjamini–Hochberg correction. Significant genes were validated by qPCR. RNA-seq data are available on request from the authors.

### 4.7. Telomere Length (TL) Assessment

Telomere length was analyzed by qPCR. Genomic DNA was isolated from frozen liver and scWAT using a lysis buffer followed by isopropanol precipitation.

Once the genomic DNA was extracted, all samples were normalized to 5ng/µL, and a calibration curve was prepared with standards of telomere (1.8 × 10^5^ to 1.8 kb) and 36B4 (2.63 × 10^5^ to 2.63 diploid copies) in 10-fold dilutions according to O’Callaghan et al. [85]. The primers used to evaluate telomer length are listed in Table S2. Samples were run in triplicate due to variability, and linearity (R^2^ > 0.99) was required. Telomere length was expressed as the telomere/36B4 ratio. 

### 4.8. Mitochondrial Biogenesis

Mitochondrial content was assessed by qPCR as the ratio of mitochondrial *cytochrome c oxidase subunit I* (*Cox1*), as a reference mitochondrial gene, to *hemoglobin beta chain complex* (*Hbb*) as a nuclear reference gene. Total DNA was extracted from liver using a lysis buffer followed by an isopropanol precipitation, and qPCR was performed using the primers listed in Table S3.

### 4.9. Protein Extraction and Western Blot Analysis

Liver tissue was homogenized in RIPA buffer with protease and phosphatase inhibitors using a pellet pestle (Z359971, Sigma-Aldrich, Saint Louis, MO, USA). Lysates were then centrifuged for 10 min at 1600 rpm and 4 °C and supernatants were recovered for analysis. Protein concentration was determined with a Pierce BCA Protein Assay Kit (23225, Thermo Scientific, Waltham, MA, USA) following manufacturer’s instructions.

To perform Western blot assays, 40 µg of protein were resolved by 10% SDS-polyacrylamide gel electrophoresis and transferred overnight to Hybond-P PVDF membrane (Millipore, Burlington, MA, USA). Membranes were blocked for 1 h at room temperature in blocking solution (Tris-HCl 50 mM pH 8, 150 mM, 5% skimmed milk, 0.5% Tween) and then blots were then incubated with primary antibodies (Table S4). After an overnight incubation at 4 °C, blots were washed and incubated for 1 h at room temperature with a fluorescent secondary antibody (goat anti-mouse green, AB_2556774, Invitrogen, Waltham, MA, USA) in blocking solution. Signals were detected at excitation wavelength of 800 nm and normalized to Vinculin levels. Relative quantification was performed with Image Studio Lite v5.2 software.

### 4.10. Statistical Analysis

All statistical analyses and standard curves were processed using GraphPad Prism version 9.3.1 (GraphPad, La Jolla, CA, USA). Data are presented as mean ± SEM. Differences between groups were assessed with two-tailed unpaired Student’s t-test; Welch’s corrections was applied when variances were unequal. A *p*-value < 0.05 was considered statistically significant.

## 5. Conclusions

In summary, our findings indicate hepatic FGF21 deficiency in aged females was associated with improved metabolic health, reflected in reduced body weight and enhanced glucose tolerance. The data presented suggest that this phenotype was accompanied by a metabolic shift toward increased fatty acid oxidation (upregulation of β-oxidation-related genes) and reduced lipogenesis (downregulation of lipogenic genes), preservation of telomere length, and reduced expression of genes linked to inflammation and fibrosis, with CHRNA4 emerging as a potential mediator of part of these effects.

Overall, our results indicate that the absence of hepatic FGF21 in female mice has an impact on the liver function in a way that may attenuate age-related metabolic deterioration. Nevertheless, further research is needed to elucidate the precise mechanisms underlying these adaptations.

Collectively, this study underscores the importance of female models in aging and metabolic research and suggests that hepatic FGF21 may exert a distinct, sex-specific role in female physiology under non-stress conditions.

## Data Availability

The raw data supporting the conclusions of this article will be made available by the authors on request.

## Supplementary Materials

The following supporting information can be downloaded at: https://www.mdpi.com/article/10.3390/ijms27010194/s1.
