# Supplementary material for: Hepatic FGF21 Deletion Improves Glucose Metabolism, Alters Lipogenic and Chrna4 Gene Expression, and Enhances Telomere Maintenance in Aged Female Mice
Source: Int J Mol Sci. 2025 Dec 24;27(1):194. doi: 10.3390/ijms27010194 (PMC12786002; doi:10.3390/ijms27010194)
Supplement: Supplementary file 1 [file ijms-27-00194-s001.zip › ijms-3976099-supplementary.pdf]

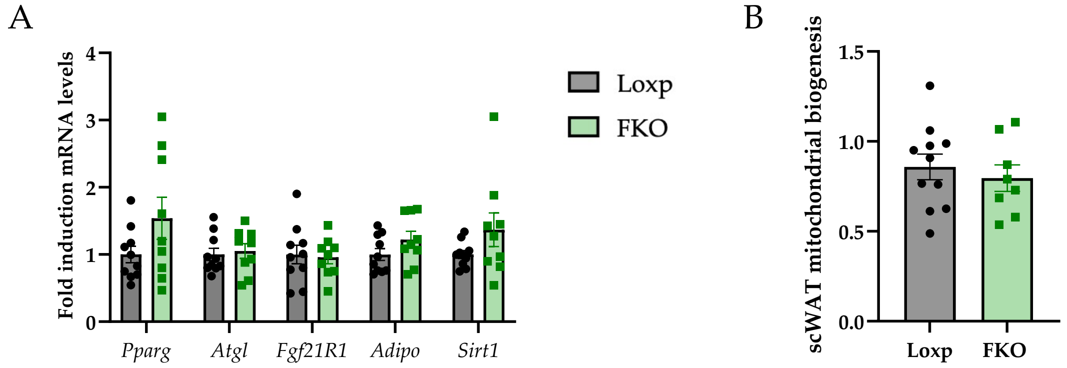

Figure S1. Hepatic FGF21 deficiency did not alter FGF21 response in scWAT. (A) Relative mRNA levels of genes related to lipid metabolism: *Pparg (Peroxisome proliferator activated receptor gamma), Atgl (Patatin-like phospholipase domain containing 2), Fgf21R1 (Fibroblast growth factor 21 receptor 1), Adipo (Adiponectin) and Sirt1 (Sirtuin 1).* (B) Ratio between genomic *Cox1 (Cytochrome c oxidase subunit I)* content and *Hbbt2 (Beta-globin).* All data are presented as mean ± SEM. p-values were determined by using a Student's T-test. LoxP: n=10; FKO: n=9.

Table S1. List of oligonucleotides used to perform qPCR.

Abbreviation	Gene	Sequence
B2m	Beta-2 microglobulin	F: ACTGATACATACGCCTGCAGAGTT<br>R: TCACATGTCTCGATCCCAGTAGA
18s	18S ribosomal RNA	F: CGGCTACCACATCCAAGGAA<br>R: GCTGGAATTACCGCGGCT
bActin	Actin, beta	F: GCTCTGGCTCCTAGCACCAT<br>R: GCCACCGATCCACACAGAGT
Acox3	Acyl-Coenzyme A oxidase 3, pristanoyl	F: GCCTCCTTCAACTCTGGGG<br>R: TCAGTTCTTCGTAGCTTCTCTAGG
Adipo	Adiponectin, C1Q and collagen domain containing	F: CAGTGGATCTGACGACACCAA<br>R: TGGGCAGGATTAAGAGGAACA
AdipoR2	Adiponectin receptor 2	F: AGCCTCTATATCACCGGAGCTG<br>R: GCTGATGAGAGTGAAACCAGATGT
Apoc2	Apolipoprotein C2 like	F: CTCTGCTGGGCACGGTGCA<br>R: GCCGCCGAGCTTTTGCTGTAC
Atgl	Patatin-like phospholipase domain containing 2	F: CGCCTCTCGAAGGCTCTCT<br>R: TGTAGCCCTGTTTGCACATCTC
Bcl6	B cell leukemia/lymphoma 6	F: GACGCACAGTGACAAACCAT<br>R: GATTGAACTGCGCTCCACAA
Cd36	CD36 molecule	F: ATGACGTGGCAAAGAACAGCT<br>R: AAGGCTCAAAGATGGCTCC
Chrebp	MLX interacting protein-like	F: CACTCAGGGAATACACGCCTAC<br>R: ATCTTGGTCTTAGGGTCTTCAGG
Chrna4	Cholinergic receptor, nicotinic, alpha polypeptide 4	F: CTCCTGCTGCTCTTAGGGAC<br>R: CATCTGGTTTTTCTCATCCACATCA
Cpt1a	Carnitine palmitoyltransferase 1a	F: AGAATCTCATTGGCCACCAG<br>R: CAGGGTCTCACTCTCCTTGC
Fas	Fatty acid synthase	F: GCTGCGGAAACTTCAGGAAAT<br>R: AGAGACGTGTCACTCCTGGACTT
Fgf21	Fibroblast growth factor 21	F: CAGTCCAGAAAGTCTCCTG<br>R: AGAAACCTAGAGGCTTTGAC
Fgf21r1	Fibroblast growth factor receptor 1	F: CTGGCAGCGATACCACCTAC<br>R: CTGGGGATGTCCAGTAGGGA
Fgf21r4	Fibroblast growth factor receptor 4	F: CTGCTTTGGGCAAGTGGTTC<br>R: TGCCAAATCCTTGTCGGAGG
G6pc	Glucose-6-phosphatase, catalytic	F: TCTGTCCCGGATCTACCTTG<br>R: GTAGAATCCAAGCGCGAAA
Gluk	Glucokinase	F: GGCGTGAAACCGCTCCTT<br>R: GTGGATGGCTCCGTGTACAA
Hmgcs2	3-hydroxy-3-methylglutaryl-Coenzyme A synthase 2	F: CCCGGTGTCCCGTCTAATG<br>R: CAGCTCGGCTCACCTTCTTT
Lpk	Pyruvate kinase liver and red blood cell	F: CATTGTGCTGACAAAGACTGG<br>R: AGCCTGTCACCACAATCACC
mtAtp6	ATP synthase 6, mitochondrial	F: ACACACCAAAAGGACGGACA<br>R: AGTGGGCGAGTGAGCTTTTT
mtCo1	Cytochrome c oxidase I, mitochondrial	F: GACTTGCAACCCTACACGGA<br>R: GATGGCGAAGTGGGCTTTTG
mtCyb	Cytochrome b, mitochondria	F: TTTATCATCGCGGCCCTAGC<br>R: ATGGGGTGGGGTGTTTAGTG
mtND3	NADH dehydrogenase 3, mitochondrial	F: TGCACGCCTACCATTCTCAA<br>R: GGTAGTGGAAGTAGAAGGGCA
Pepck	Phosphoenolpyruvate carboxykinase 1, cytosolic	F: CTTTGGTGGCCGTAGACCTG<br>R: GATGATCTTGCCCTTGTGTTCTG
Pgc1a	Peroxisome proliferative activated receptor, gamma, coactivator 1 alpha	F: AACCACACCCACAGGATCAGA

		R: CTCTTCGCTTTATTGCTCCATGA
Ppara	Peroxisome proliferator activated receptor alpha	F: GATTCAGAAGAAGAACCGGAACA
		R: GCGAATTGCATTGTGTGACAT
Pparg	Peroxisome proliferator activated receptor gamma	F: GCATCAGGCTTCCACTATGGA
		R: AATCGGATGGTTCTTCGGAAA
Prdx1	Peroxiredoxin 1	F: AATGCAAAAATTGGGTATCCTGC
		R: CGTGGGACACACAAAAGTAAAGT
Scd1	Stearoyl-Coenzyme A desaturase 1	F: CTTGCGGATCTTCCTTATCATT
		R: GATCTCGGGCCCATTCG
Sirt1	Sirtuin 1	F: GACGCTGTGGCAGATTGTTA
		R: GGAATCCCACAGGAGACAGA
Srebp1c	Sterol regulatory element binding transcription factor 1	F: GGAGCCATGGATTGCACATT
		R: GGCCCGGGAAGTCACTGT
Terc	Telomerase RNA component	F: TCATTAGCTGTGGGTTCTGGT
		R: TGGAGCTCCTGCGCTGACGTT
Tert	Telomerase reverse transcriptase	F: GGATTGCCACTGGCTCCG
		R: TGCCTGACCTCCTCTTGTGAC
Tgfb	Transforming growth factor, beta 1	F: ACGTCACTGGAGTTGTACGG
		R: GGGCTGATCCCGTTGATTTC

Table S2. List of oligonucleotides and standards used for TL analysis.

Abbreviation	Sequence	
Genomic 36B4	F: CAGCAAGTGGGAAGGTGTAATCC	R: CCCATTCTATCATCAACGGGTACAA
Telomere	F: CGGTTTGTTTGGGTTTGGGTTTGGGTT TGGGTTTGGGTT	R: GGCTGCCTTACCCTTACCCTTACCCTTACC CTTACCCT
Telomere standard	14 tandem copies of TTAGGG	
36B4 standard	CAGCAAGTGGGAAGGTGTAATCCGTCTCCACAGACAAGGCCAGGACTCGTTTGTACCCG TTGATGATAGAATGGG	

Table S3. List of oligonucleotides used for mitochondrial biogenesis analysis.

**Abbreviation**	**Gene**	**Sequence**
Cox1	Cytochrome c oxidase subunit I	F: CTGAGCGGGAATAGTGGGTA<br>R: TGGGGCTCCGATTATTAGTG
Hbb	Hemoglobin beta chain complex	F: GCACCTGACTGATGCTGAGAA<br>R: TTCATCGGCGTTCACCTTTCC

Table S4. List of antibodies used for Western Blot analysis.

Antibody	Dilution	Company	Reference
SDHA	1:10000	Invitrogen	AB_10838019
ATP5A1	1:1000	Invitrogen	AB_2532234
MTCO2	1:1000	Abcam	ab110258
VINCULIN	1:1000	Santa Cruz	sc-73614
Goat anti-Mouse IgG	1:10000	Invitrogen	AB_2556774
